# Breath of relief: Transforming pediatric asthma care with telemedicine‐guided exercises

**DOI:** 10.1002/clt2.70049

**Published:** 2025-03-24

**Authors:** Betul Gemici Karaaslan, Hikmet Ucgun, Meltem Kaya, Gokce Nuran Cengiz, Sueda Ozturk, Ozge Barut, Zeynep Korkut, Sezin Aydemir, Zeynep Meric, Birol Topcu, Hilal Denizoglu Kulli, Haluk Cokuğras, Ayca Kiykim

**Affiliations:** ^1^ Department of Pediatric Immunology and Allergy School of Medicine Istanbul University‐Cerrahpaşa Istanbul Turkey; ^2^ Department of Physiotherapy and Rehabilitation Biruni University Istanbul Turkey; ^3^ Department of Physiotherapy and Rehabilitation Atlas University Istanbul Turkey; ^4^ Department of Pediatrics School of Medicine Istanbul University‐Cerrahpaşa Istanbul Turkey; ^5^ Cerrahpasa School of Medicine Istanbul University‐Cerrahpaşa Istanbul Turkey; ^6^ Department of Biostatistics Tekirdag Namik Kemal University Tekirdag Turkey

**Keywords:** asthma, breathing exercises, respiratory rehabilitation

## Abstract

**Background:**

Alternative non‐pharmacological strategies such as breathing exercises can be used in combination with pharmacological treatments.

**Objective:**

The aim of this randomized, controlled, single‐blind study was to investigate the effectiveness of breathing exercises in asthma patients on respiratory function, symptom control and quality of life.

**Methods:**

We enrolled pediatric asthma patients who were eligible and motivated for the study and randomly assigned them to either the exercise group (EG) or the control group (CG). The CG received a postural exercise program, while the EG received a breathing exercise program. At baseline and after 12 weeks, respiratory function (FEV1‐FVC‐FEV1/FVC‐PEF), symptom control (using asthma control test, asthma control questionnaire, global initiative for asthma symptom control assessment), quality of life (using pediatric asthma quality of life questionnaire), breath‐holding test (BHT) and sit‐to‐stand test (30sSTS) were assessed and compared.

**Results:**

One hundred twelve patients were randomized, and 99 (*n* = 51 EG, *n* = 48 CG) completed the 12‐week study. Baseline data were also similar in both groups. After 12 weeks, FEV1, Peak expiratory flow (by spirometry and peak flow meter) and BHT were significantly better in EG than in CG (*p* = 0.01 and *p* = 0.007 and *p* = 0.005, respectively). Asthma Control Test and GINA symptom control tool values were also significantly better in both groups.

**Discussion:**

Our participants were children with mild to moderate asthma. We conclude that our results show that breathing exercises can be an effective intervention for children with partially controlled asthma with FEV1,PEF, and BHTs.

## 1 INTRODUCTION

The high prevalence of asthma in children is a public health problem as it leads to healthcare costs, frequent hospitalizations, multiple medications, and missed school days.^1^ Asthma is a chronic inflammatory disease that can lead to structural and functional changes in bronchial structure.^1^, ^2^ Poor asthma control leads to numerous visits to the doctor/emergency services and repeated hospitalizations despite pharmacological treatment.^3^ The main aim of asthma treatment is to control symptoms and preserve optimal respiratory function.^4^ Pharmacological treatment focuses on achieving symptom control with the minimum effective dose of medication while minimizing adverse effects.^5^ In addition to pharmacological treatments, complementary non‐pharmacological strategies, including regular physical activity, avoidance of occupational exposures, mitigation of medication‐induced exacerbations, smoking cessation, and reduction of indoor allergens, can play a supportive role in asthma management.^4^

Breathing exercises, including the Buteyko method, the Papworth method, or yoga, can be a non‐pharmacological treatment. Pulmonary rehabilitation is particularly recommended for individuals with chronic lung diseases who experience diminished aerobic capacity and muscle weakness.^6^ Controlling overbreathing or hyperventilation symptoms facilitates the modification of maladaptive breathing patterns through structured breathing exercises.^1^, ^7^ These exercises aim to normalize CO2 levels, which can mitigate bronchospasm, reduce dyspnea, and improve overall respiratory function.^8^

Evidence supporting non‐pharmacological strategies like breathing exercises remains limited because of the lack of high‐quality randomized controlled trials (RCTs). In addition, systematic reviews of breathing and/or relaxation exercises in adults and children with asthma have reported improvements in symptoms, quality of life, and/or psychological measures, but without effects on lung function in adults and without conclusive evidence for the benefits of breathing exercises in children.^1^, ^7^, ^9^ Furthermore, the Cochrane Database Syst Rev and the global initiative for asthma (GINA) 2022 report highlighted methodological differences between studies and inadequate reporting of both methodologies and outcomes in the majority of the research.^1^, ^2^ Despite these limitations, GINA 2022 concluded that breathing exercises may be considered an adjunct to conventional asthma management strategies for improving symptoms and quality of life. However, they do not enhance lung function or reduce the risk of exacerbations (Evidence A).^4^

This randomized‐controlled, single‐blind study was designed to evaluate the effectiveness of breathing exercises in asthma patients in improving lung function (FEV1, FVC, FEV1/FVC, Peak expiratory flow (PEF)), symptom control (using the Asthma Control Test (ACT), the Asthma Control Questionnaire (ACQ), the GINA Symptom Control Assessment) and quality of life (using the Pediatric Asthma Quality of Life Questionnaire (PAQLQ)). This study aimed to explore the potential role of breathing exercises as a complementary approach to conventional asthma management.

## 2 METHODS

### 2.1 Experimental design

The effects of breathing exercises through telemedicine in patients aged 8–18 years with a diagnosis of asthma (PHASTER) was a parallel, 12‐week, randomized‐controlled, single‐blinded study conducted at Istanbul University‐Cerrahpasa in the Pediatric Immunology and Allergy Clinic. The Ethics Committee of Istanbul University‐Cerrahpasa approved the study protocol on 13.07/13/2021‐E−29430533‐604.01.01–139431, and the study was included in ClinicalTrials.gov (NCT05749237). We followed the ethical guidelines and obtained informed consent from all participants.

### 2.2 Study participants

**FIGURE 1:**
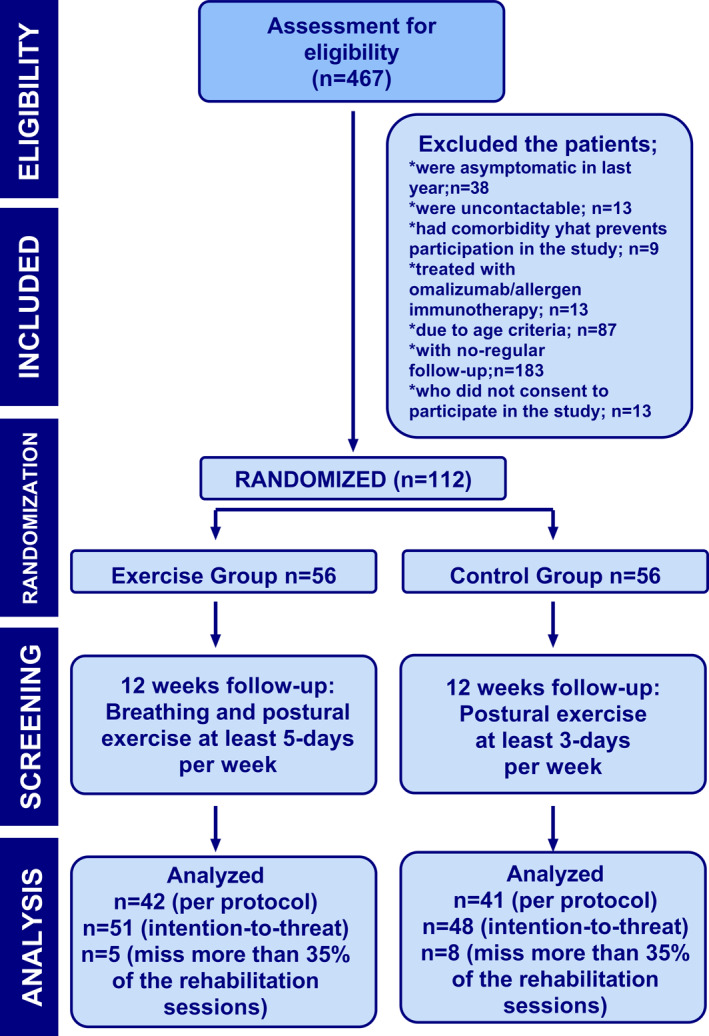
Summary of patients who entered, randomized, and withdrew from the trial.

Patients aged 8–18 years with physician‐diagnosed mild to moderate asthma were recruited for the study. The study diagram, outlining the flow of participants through the trial, is illustrated in Figure 1 and the exclusion criteria were specified in the eSupplementary Material.

### 2.3 Study setting

We enrolled pediatric asthma patients from Istanbul University‐Cerrahpasa. We started the study in January 2023, recruitment ended in March 2023, and the last follow‐up was conducted in July 2023. Research assistants contacted all interested individuals via Zoom, WhatsApp, and telephone to facilitate accessibility. Patients identified as suitable and motivated for the study were randomly assigned to either the exercise group (EG) or the control group (CG). Patients were invited to the first appointment where they were given general information about asthma self‐management and the purpose and content of the study were explained. They were asked to give informed consent and then complete a respiratory function test, a 30‐s sit‐to‐stand test (30sSTS), a breath‐hold test (breath‐holding test (BHT)) and scales for asthma control (ACT, ACQ, GINA Symptom Control Tool [GINA‐SCT]) and quality of life (PAQLQ). (See in Supporting Information S1: eTable S1). The CG received a postural exercise program and the EG a breathing exercise program (as detailed in eTable S2).

### 2.4 Exercise intervention and follow‐up

Asthma control was assessed at baseline using validated questionnaires, respiratory function tests (conducted in accordance with American Thoracic Society (ATS)/European Respiratory Society (ERS) recommendations), and a 1‐week PEF assessment with a peak flow meter. During the baseline clinical visit, a physiotherapist instructed both groups on postural exercises, and the exercise instructions were supplemented with video modules delivered via telemedicine for repeated viewing. Additionally, the EG received specific training on breathing exercises during the same visit.

The CG followed a postural exercise program 3 days per week for 12 weeks, while the EG performed a breathing exercise program 5 days per week alongside postural exercises. Both groups accessed pre‐recorded modules to guide their exercises throughout the study. The postural exercise program included three stretching exercises and relaxation positions to enhance rib cage mobility and chest wall muscle flexibility. The breathing exercise program incorporated lip breathing, diaphragmatic breathing, and lateral rib breathing to improve asthma control, respiratory function, and dyspnea awareness.

During the 12‐week follow‐up period, the researchers asked both CGs and EGs to continue their exercise program and to measure PEF regularly via telemedicine. All participants were asked to record in their diaries the duration, intensity and type of exercise performed each day. They were also asked to record any asthma symptoms and activity limitations in their diaries. Participants also recorded weekly PEF measurements.

At the end of the 12‐week period, baseline measurements, including spirometry, 30sSTS, BHT and all questionnaires, were repeated during the clinical visit.

### 2.5 Outcomes

The primary outcome was assessed via spirometry‐based respiratory function tests. Changes in FEV1, FVC, FEV1/FVC, and PEF were recorded for both groups. According to the ERS and ATS guidelines, z‐scores were used as the primary method for analyzing spirometry data.

Morning PEF measurements were performed at home using a peak flow meter prior to inhaled corticosteroids (ICS)/inhaled corticosteroids‐long acting beta 2 agonists (ICS‐LABA) administration. The measurement periods were analyzed and the mean value of PEF measurements was calculated every 4 weeks.

Functional capacity was evaluated using the 30sSTS test. The 30sSTS test was performed according to Bohannon's 2012 guidelines.^10^ Perception of dyspnea was assessed using the BHT, which measures the duration a patient can hold their breath under controlled conditions.^11^

The primary endpoint was the mean change in FEV1 and PEF measured by spirometry. All spirometry parameters were analyzed using an intention‐to‐treat approach with a minimum compliance threshold of 65% for the intervention program. Patients who achieved at least 90% compliance were classified as the “full program cohort,” and their outcomes were further examined using a per‐protocol analysis.

Secondary outcomes included mean changes in ACT, ACQ, GINA‐SCT, PAQLQ, 30sSTS, BHT, and PEF as measured by the peak flow meter. These outcomes were analyzed using the per‐protocol approach. Additionally, overall asthma control was assessed using the ACT, ACQ, and GINA‐SCT. These validated tools are widely used to evaluate asthma control (see Supporting Information S1: eTable S1 for details on these questionnaires).^12^, ^13^, ^14^, ^15^ See detailed methods in eMethods.^11^, ^16^, ^17^, ^18^, ^19^, ^20^, ^21^

## 3 RESULTS

Among 467 patients screened, 125 met the inclusion criteria, and 13 declined to participate in the study. A total of 112 patients were randomized, of whom 99 (*n* = 51 EG, *n* = 48 CG) completed the 12‐week study. Of these, 83 patients were categorized as the “full program cohort,” comprising individuals from both groups who adhered to the program consistently for the full 12 weeks. Thirteen patients were excluded because they could not be reached during the study period. All participants were able to contact the researchers when needed.

Baseline characteristics, including age, sex, body mass index, spirometry parameters, and scores from ACT, ACQ, GINA‐SCT, PAQLQ, BHT, and 30sSTS, were comparable between the two groups (detailed data available in eTable S3 and eTable S4 of the eSupplementary material). The mean age of the EG was 158.35 ± 34.2 months, while the CG had a mean age of 150.59 ± 29.74 months.

### 3.1 Respiratory function test results and measurement of Peak expiratory flow

Respiratory functions were assessed using spirometry. In the EG, the mean baseline values for FEV1, FEV1/FVC, PEF, and FVC were 98.92 ± 14.57, 102.78 ± 10.35, 85.74 ± 15.64, and 99.44 ± 20.32, respectively. After the 12‐week intervention period, these values increased to 103.5 ± 14.75, 106.04 ± 9.89, 95.68 ± 13.52, and 97.24 ± 13.15, respectively. Intention‐to‐treat analysis revealed significant improvements in FEV1, FEV1/FVC, and PEF at 12 weeks compared with baseline (*p* = 0.01, *p* = 0.007, and *p* < 0.0001, respectively). No significant change was observed in FVC within the EG (*p* = 0.384).

In the CG, the mean baseline values for FEV1, FEV1/FVC, PEF, and FVC were 100.71 ± 13.17, 102.71 ± 9.23, 89.77 ± 15.17, and 97.67 ± 14.07, respectively. After the 12‐week follow‐up period, these values were 101.21 ± 12.6, 104.82 ± 9.42, 93.4 ± 19.09, and 96.3 ± 13.7, respectively. No significant changes in FEV1, FEV1/FVC, or PEF were observed in the CG (*p* = 0.742, *p* = 0.138, and *p* = 0.194, respectively). Similarly, FVC did not show significant differences in either group (EG: *p* = 0.384, CG: *p* = 0.21).

These findings highlight a significant improvement in respiratory function parameters in the EG compared with the CG, particularly for FEV1, FEV1/FVC, and PEF. Full details are presented in Table 1 and eFigures S1.In line with ERS/ATS guidelines recommendations, z‐score calculations were performed to compare baseline and post‐intervention spirometric outcomes between the EG and the CG. In the EG, the baseline FEV1 z‐score had a median value of −0.24 (interquartile range (IQR) 25th–75th: −0.79; 1.27). After the 12‐week intervention, the median FEV1 z‐score improved to 0.40 (IQR 25th–75th: −0.33; 0.96), showing a statistically significant change (*p* = 0.014). In the CG, the baseline FEV1 z‐score median value was −0.15 (IQR 25th–75th: −1.37; 0.83). At the end of the 12 weeks, the median FEV1 z‐score was observed to be 0.07 (IQR 25th–75th: −0.56; 0.86), which was not statistically significant (*p* = 0.161) (eFigure S2).

**TABLE 1:** The mean values for outcome measures at baseline and 3‐month follow‐up (*n* = 99) Intention‐to‐threat analysis.

		Baseline	At 12 weeks	Change from baseline	
		Mean, SD	Mean, SD	Δ	*p* value
FEV1 (%)	CG	100.71 (13.17)	101.21 (12.6)	0.5	0.742
	EG	98.92 (14.57)	103.5 (14.75)	4.58	**0.01**
FVC (%)	CG	97.67 (14.07)	96.3 (13.7)	−1.36	0.21
	EG	99.44 (20.32)	97.24 (13.15)	−2.2	0.384
FEV1/FVC (%)	CG	102.71 (9.23)	104.82 (9.42)	2.1	0.138
	EG	102.78 (10.35)	106.04 (9.89)	3.62	**0.007**
PEF (spirometry) (%)	CG	89.77 (15.17)	93.4 (19.09)	3.66	0.194
	EG	85.74 (15.64)	95.68 (13.52)	9.94	**<0.0001**

*Note*: Bold indicates significant *p* values.

Abbreviations: CG, Control Group; EG, Exercise Group; FEV1, forced expiratory volume in 1 s [%]; FVC, forced vital capacity; PEF, Peak expiratory flow; Δ, mean between‐difference.

The per‐protocol analyses for the “full program cohort” included *n* = 42 patients in the EG and *n* = 41 patients in the CG. In the EG group, FEV1 and PEF were significantly increased after 12 weeks compared with baseline (*p* = 0.045 and *p* = 0.001, respectively). In CG, there was no significant change in FEV1 and PEF between baseline and 3 months (*p* = 0.206 and *p* = 0.181, respectively). Similarly, no significant differences were observed in FVC and FEV1/FVC between the groups (EG *p* = 0.666 and CG *p* = 0.369) (Table 2).

**TABLE 2:** Mean values for outcome measures at baseline and 3‐month follow‐up (*n* = 83) Per‐protocol analysis.

		Baseline	At 12th week	Change from baseline	
		Mean. SD	Mean. SD	Δ	*p* value
FEV1 (%)	CG	98.05 (11.5)	99.88 (11.79)	1.85	0.206
	EG	99.65 (15.4)	103.68 (15.12)	4.03	**0.045**
FVC (%)	CG	95.18 (12.66)	94.13 (12.4)	−1.05	0.369
	EG	98.7^22^	97.41^14^	−1.29	0.666
PEF (spirometry) (%)	CG	87.95 (14.16)	91.97 (18.5)	4.02	0.181
	EG	86.22 (15.17)	96.34 (13.12)	10.12	**0.001**
PEF (peak flow‐meter)	CG	355.5 (115)	366.3 (101)	10.8	0.025
	EG	347.9 (132.8)	393.6 (122.6)	45.7	**<0.0001**
BHT	CG	27.62 (10.65)	27.37 (15.25)	−0.25	0.274
	EG	27.37 (15.25)	35.36 (17.75)	7.99	**0.005**
30sSTS	CG	17.7 (3.58)	18.8 (4.12)	1.1	0.387
	EG	18.84 (3.93)	19.52 (3.86)	0.68	0.245

*Note*: Bold indicates significant *p* values.

Abbreviations: 30sSTS, 30 s sit‐to‐stand test; BHT, breath‐holding time test; CG, Control Group; EG, Exercise Group; FEV1, forced expiratory volume in 1 s [%]; FVC, forced vital capacity; PEF, Peak expiratory flow; Δ, mean between‐difference.

We hypothesize that the differences between the per‐protocol and intention‐to‐treat analyses may be due to the fact that the per‐protocol analysis included only those patients who were fully compliant with the intervention, whereas the intention‐to‐treat analysis included all patients even with lower compliance.

### 3.2 Peak flow meter and functional test results

Peak expiratory flow measurements with the peak flow meter remained relatively stable in the CG but showed a significant increase in the EG. The median change (IQR in PEF values after 12 weeks was −45.8 (−65.3 to −26.3) in the EG and −27.85 (−51.9 to −3.78) in the CG (*p* = 0.001 and *p* = 0.025, respectively).

**FIGURE 2:**
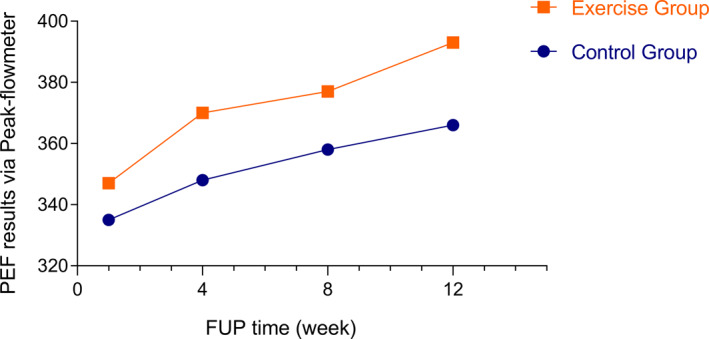
Mean change of 1w, 4w, 8w, and 12w of Peak expiratory flow (PEF) levels during the study, 1w‐12w changes: *p* = 0.025 in control group (CG), *p* < 0.0001 in exercise group (EG) (*n* = 83).

In the EG, significant changes in PEF were observed during all analyzed intervals (1–4 weeks, 1–8 weeks, 4–12 weeks, and 8–12 weeks; all *p* < 0.05). In contrast, no significant changes were detected in the CG across the same time intervals (Figure 2).

**BHT:** The mean baseline breath‐holding time was 27.37 ± 15.25 s for EG and 27.62 ± 10.65 s for CG (*p* > 0.05). After the intervention, the mean value of breath holding time at EG increased significantly to 35.36 ± 17.75 s (*p* = 0.005), while the mean value of BHT at CG remained unchanged (27.37 ± 15.25 s, *p* = 0.274) (Table 2).

**30sSTS:** The baseline levels of 30sSTS were nearly similar for both groups (EG: 18.84 ± 3.93, CG 17.7 ± 3.58). After the intervention, the changes were −0.68 in the EG and −1.1 in the CG, neither of which were statistically significant (respectively *p* = 0.245 and *p* = 0.387). Detailed changes in respiratory function are presented in Tables 1 and 2.

### 3.3 Changes in the results of questionnaires

**ACT:** The mean baseline ACT scores were similar between the groups (CG: 19.94 ± 3.46; EG: 19.5 ± 3.92). Post‐intervention, significant improvements were observed in ACT scores within both groups (CG: 21.97 ± 3.49, *p* = 0.001; EG: 22.65 ± 2.6, *p* < 0.001). Additionally, the EG group showed a notable difference during the 12‐week follow‐up period, with a large effect size observed (Table 3).

**TABLE 3:** Mean values for questionnaire outcome measures at baseline and 3‐month follow‐up (*n* = 83) Per protocol analysis.

		Baseline	At 12th week	Change from baseline	*p* value
		Mean, SD	Mean, SD	Δ	
ACT	CG	19.94 (3.46)	21.97 (3.49)	2.03	**0.001**
	EG	19.5 (3.92)	22.65 (2.6)	3.15	**<0.0001**
ACQ	CG	1.12 (0.7)	0.69 (0.84)	−0.43	**0.001**
	EG	0.9 (0.74)	0.45 (0.59)	−0.45	**0.001**
GINA‐SCT	CG	1.2 (0.99)	0.74 (1.01)	−0.45	**0.024**
	EG	1.11 (1.26)	0.53 (0.97)	−0.57	**0.003**
PAQLQ‐total	CG	5.65 (0.84)	6.04 (0.97)	0.39	**0.004**
	EG	5.67 (1.1)	6.27 (0.74)	0.6	**<0.0001**
PAQLQ‐symptoms	CG	5.36 (1.01)	5.75 (1.16)	0.39	**0.001**
	EG	5.44 (1.22)	6.13 (0.97)	0.69	**<0.0001**
PAQLQ‐activity limitation	CG	5.42 (1.15)	6.12 (1.07)	0.7	**0.025**
	EG	5.47 (1.23)	6.25 (1.02)	0.78	**0.002**
PAQLQ‐emotional function	CG	6.17 (0.94)	6.35 (0.95)	0.18	0.218
	EG	6.07 (1.2)	6.47 (0.67)	0.4	**0.022**

*Note*: Bold indicates significant *p* values.

Abbreviations: ACT, asthma control test; ACQ, asthma control questionnaire; CG, Control Group; EG, Exercise Group; GINA‐SCT, GINA symptom control tool; PAQLQ, pediatric asthma quality of life questionnaire; Δ, mean between‐difference.

**ACQ:** The mean baseline ACQ scores were 0.9 ± 0.74 for the EG and 1.12 ± 0.7 for the CG. A significant difference in ACQ scores was observed at the 3‐month follow‐up in both groups, with a moderate effect size (*p* = 0.001 for both groups) (Table 3).

**GINA‐SCT:** The mean baseline GINA‐SCT scores were 1.11 ± 1.26 for the EG and 1.2 ± 0.99 for the CG. Post‐intervention, GINA‐SCT scores improved significantly in both groups, with greater changes observed in the EG. The post‐intervention mean GINA‐SCT scores were 0.53 ± 0.97 for the EG (*p* = 0.003) and 0.74 ± 1.01 for the CG (*p* = 0.024) (Table 3).

**PAQLQ:** Both groups demonstrated significant improvements after 12 weeks; however, the mean difference was significantly greater in the EG group (0.6) compared to the CG CG (0.39), with a larger effect size (*p* < 0.0001 to *p* = 0.004; Table 3).

The mean PAQLQ symptom score improved from 5.44 to 6.13 in the EG and from 5.36 to 5.75 in the CG. Similarly, the activity limitation subscale scores increased from 5.47 to 6.25 in the EG and from 5.42 to 6.12 in the CG. The mean emotional function scores at baseline were 6.07 ± 1.2 for the EG and 6.17 ± 0.94 for the CG. After the intervention, these scores improved to 6.47 in the EG and 6.35 in the CG.

Subscale analysis revealed that symptoms and activity limitation improved significantly in both groups after 12 weeks, whereas emotional function showed a significant improvement only in the EG (*p* = 0.022). Detailed changes in questionnaire scores are summarized in Table 3.

### 3.4 Change in clinical characteristics

Clinical changes were evaluated using both questionnaires and modifications in treatment steps. As detailed in the previous section, the questionnaire results showed significant improvements in certain areas. However, no significant differences were observed in the treatment steps at baseline (*p* = 0.34) (Supporting Information S1: eTable S3):

In the CG:

- 18 patients received regular treatment with inhaled corticosteroids (ICS).
- 21 patients received regular treatment with ICS combined with a long‐acting beta‐agonist.
- 7 patients were treated with ICS‐LABA on an as‐needed basis.
- 2 patients were treated with ICS on an as‐needed basis.
  In the EG:
- 18 patients received regular treatment with ICS.
- 28 patients received regular treatment with ICS‐LABA.
- 4 patients were treated with ICS‐LABA on an as‐needed basis.
- 1 patient was treated with ICS on an as‐needed basis.

**FIGURE 3:**
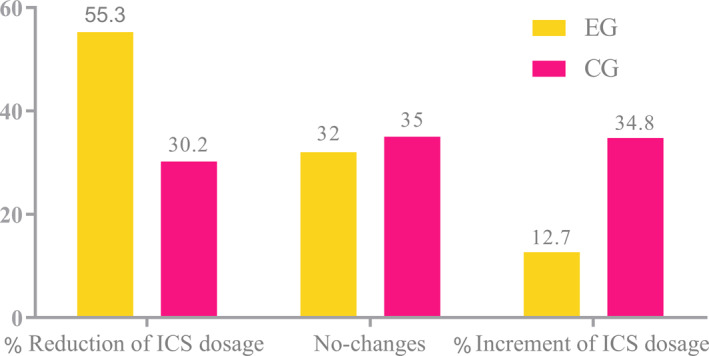
Treatment changes after intervention in both groups (*n* = 99).

Inhaled corticosteroids dose adjustments were conducted in accordance with the GINA 2022 guidelines. Treatment steps remained unchanged for most patients during the study, and the 12‐week treatment schedules were determined by blinded physicians. At the end of the study, treatment step‐up was required in 15 patients from the CG group, compared to only 6 patients in the EG group, representing 34.8% and 12.7%, respectively (*p* = 0.029, excluding patients with no changes). Conversely, treatment was downgraded (step‐down) significantly more often in the EG group (*n* = 26, 55.3%) compared to the CG group (*n* = 13, 30.2%; *p* = 0.01). The proportion of patients with unchanged treatment was similar (EC *n* = 15, CG *n* = 15) (Figure 3).

In the CG group, five patients who were already using treatment (ICS or ICS‐LABA) on an as‐needed basis (rather than receiving regular treatment) were evaluated. Among these, three showed improvement in their respiratory status compared with baseline, and as a result, their clinical follow‐up intervals were extended. In the EG, all four patients using treatment on an as‐needed basis demonstrated improvements, leading to an extension of their follow‐up intervals.

There were no significant differences between the groups in the number of exacerbations during the 3‐month study period. The mean number of exacerbations was 0.41 ± 0.60 in the EG and 0.58 ± 0.72 in the CG.

In terms of individual outcomes, 31 participants in the EG and 25 in the CG experienced no exacerbations at all. Among those who experienced exacerbations, fewer than three exacerbations occurred in 13 patients in the EG and 15 patients in the CG, while three or more exacerbations occurred in 3 patients in the EG and 6 patients in the CG.

## 4 DISCUSSION

In this single‐blind, RCT, we evaluated the effects of a structured breathing exercise program on symptom control, quality of life, and lung function in pediatric asthma patients using telemedicine. Our findings demonstrated that breathing exercises, performed by patients five days per week under the guidance of trained physiotherapists, led to significant improvements in lung function and quality of life over a 12‐week period.

Recent clinical guidelines recommend breathing exercises as an adjunct treatment to improve quality of life in patients with mild to moderate asthma with persistent impaired control. However, their impact on respiratory function remains unclear.^11^ Previous studies have reported positive effects on asthma control and quality of life, as well as a reduction in medication use, whereas lung function remained largely unchanged.^22^, ^23^, ^24^, ^25^, ^26^, ^27^, ^28^, ^29^

Dysfunctional breathing often results in hyperventilation and hyperinflation. Breathing exercises aim to establish an appropriate breathing pattern and normalize CO2 levels, which in turn helps reduce hyperventilation, bronchospasm, and dyspnea.^8^, ^23^ The differences in improvement in respiratory function between adults and children may be attributed to the greater potential for reversing asthma‐related functional and structural changes in the respiratory system in children, likely due to the shorter duration of the disease.

The effect of breathing exercises in children with asthma was systematically reviewed in 2016 with three studies meeting the inclusion criteria.^1^, ^30^ These studies focused on diaphragmatic breathing with side rib breathing, mouth‐to‐lip breathing, or endurance training. The severity of disease among patients varied, and the primary outcomes assessed were quality of life, asthma symptoms, and adverse events. However, the meta‐analysis found that differences in primary outcomes were not significant with the exception of one study in which PEF improved after the intervention.^30^

Subsequent studies suggest that, unlike earlier findings in adult patients, pulmonary function appears to respond to breathing exercises in many children. The RCT by Elnaggar and Shendy showed significant improvements in serum IgE levels, respiratory function, and asthma awareness at the 3‐month follow‐up.^31^ Similarly, a study comparing the Buteyko breathing technique (BBT) and yoga demonstrated notable improvements in lung function and functional capacity as measured by a six‐minute walk test.^32^

Two additional studies further supported previous studies, emphasizing the beneficial effects of breathing exercises on asthma control and functional outcomes.^33^, ^34^ We found a significant effect on physiological variables, including lung function.^35^, ^36^, ^37^ Specifically, a significant difference was observed between the two groups in the changes in FEV1 and PEF, with a more pronounced improvement in PEF measurements. Our findings underscore the improvement in lung function observed in the EG following the intervention, as reflected by z‐score analysis, while the CG did not exhibit significant changes. The significantly higher BHT levels observed in the EG further corroborated the study findings, supporting the positive impact of the intervention on respiratory function and control.

Previous studies have identified the 30‐s sit‐to‐stand test (30sSTS) as a reliable alternative for assessing exercise capacity in various lung diseases, including chronic obstructive pulmonary disease, cystic fibrosis, and lung transplantation.^38^, ^39^ He ability to perform sit‐to‐stand tasks has been shown to be a strong predictor of functional capacity. Most studies report significant correlations between the 30sSTS and the 6‐min walk test, a commonly used measure of functional exercise capacity.^38^, ^39^ In addition, the 30sSTS is relatively safe to perform due to the lower cardiorespiratory workload. In our study, there was no significant difference after the exercise intervention in both groups for 30sSTS.

Although the improvements in the CG were less pronounced than those in the EG, notable enhancements were still observed in weekly average PEF measurements (assessed via the PEFmeter) as well as in ACT, GINA‐SCT, PAQLQ‐total, and the symptoms and activity limitations subscales. Both groups showed comparable improvements in ACQ scores. The accessory respiratory muscles play a key role in maintaining posture. Therefore, the postural exercises provided to the CG may have contributed to symptom control and improved certain respiratory function parameters by strengthening these muscles.^40^ This effect likely explains the observed changes in PEF values and asthma control scores in CG patients.

In the secondary outcomes, a significant difference was observed between the two groups regarding changes in treatment steps based on the GINA guidelines. The use of ICS decreased significantly in the EG, while it remained increased in the CG. Previous studies have similarly reported that breathing exercises can reduce medication use.^26^, ^28^, ^29^ McHugh et al. described a case series of eight children using the BBT, showing a reduction in beta‐2 agonists and ICS use, though no lung function data were provided.^26^

Most previous studies did not collect detailed data on asthma exacerbations, nocturnal symptoms and emergency department visits before the last year. Unlike these studies, we evaluated asthma exacerbations, daytime and nocturnal symptoms and beta‐2 agonist use through ACT, ACQ and GINA‐SCT, which reflect disease severity. Both groups showed significantly improved disease control on these questionnaires; however, the ACT and GINA‐SCT scores in the EG group were significantly better than those in the CG group with a higher effect size. Although ACQ scores improved similarly in both groups, treatment adjustments were guided by a combination of respiratory function tests, questionnaire results, and clinical visits.

Interestingly, while CG showed improvements in self‐reported ACT, ACQ, and GINA‐SCT scores, no changes in objective respiratory function were observed. This positive change may be attributed to the relaxation effect and improved breathing pattern resulting from relaxation exercises.

The asthma exacerbation rate during the study was similar in both groups. Although the number of patients without asthma attacks was higher in the EG group, this difference did not reach statistical significance.

The quality of life in asthma has already been investigated in a limited number of studies. In children, Yilmaz et al. reported that quality of life improved significantly in the intervention group at baseline and 1 month later compared to the CG.^41^ Similarly, the most recent study in adults demonstrated that asthma‐related quality of life improved after 6 months and persisted after 12 months in severe asthma with a more favorable effect observed in mild to moderate asthma in adults.^8^, ^23^, ^42^ In our study, both groups showed significant improvements in the overall PAQLQ score and the activity limitation and symptoms subscales, with the EG group showing a significantly greater change. Notably, a significant improvement was observed in the Emotions subscale in the EG group, whereas no statistically significant change was found in the CG group. We attribute the improvements in PAQLQ total and subscale scores in the CG group to the opportunity patients had to consult with researchers when they needed support.

Our study has some limitations. While we were unable to blind the researchers and physiotherapists to the intervention, which might have introduced non‐specific, context‐dependent effects in the EG participants themselves were blinded. All data collection, analysis and patient follow‐up by clinicians during assignment were conducted in a blinded manner. In addition, no long‐term effects of the breathing exercises were assessed in this study. Furthermore, the constant contact between patients and researchers during the study, which allowed patients to approach the researchers when needed — unlike the patient visits prior to the study — may have introduced a bias, potentially influencing the observed improvements in quality of life scores. Additionally, it should be acknowledged that pulmonary function test parameters can improve with repeated practice, which may have contributed to the improvements observed in the experimental group independent of the intervention itself. This potential effect should be taken into account when interpreting the findings.

In summary, our participants were children with mild to moderate asthma, allowing our results to be applicable to a significant portion of the pediatric asthma population. However, our study did not include children with severe or uncontrolled asthma, limiting the generalizability to this subgroup. We conclude that our results may provide evidence that breathing exercises can be an effective intervention for children with partially controlled asthma with FEV1, PEF and BHTs. Future studies should focus on investigating when and why children benefit from breathing exercises.

## AUTHOR CONTRIBUTIONS

**Betul Gemici Karaaslan**: Investigation; writing—original draft; conceptualization; methodology; visualization; software; data curation; validation. **Hikmet Ucgun**: Conceptualization; investigation; methodology; data curation; writing—original draft. **Meltem Kaya**: Conceptualization; investigation; writing—original draft; methodology; data curation. **Gokce Nuran Cengiz**: Investigation; data curation. **Sueda Ozturk**: Investigation; data curation. **Ozge Barut**: Investigation; data curation. **Zeynep Korkut**: Investigation; data curation. **Sezin Aydemir**: Investigation and data curation. **Zeynep Meric**: Investigation; data curation. **Birol Topcu**: Software; formal analysis; visualization. **Hilal Denizoglu Kulli**: Conceptualization; investigation; methodology; data curation; writing—original draft; writing—review and editing; formal analysis; supervision. **Haluk Cokuğras**: Funding acquisition; writing—review and editing; project administration; resources; validation. **Ayca Kiykim**: Conceptualization; investigation; funding acquisition; methodology; validation; visualization; writing—review and editing; formal analysis; project administration; data curation; supervision; resources.

## CONFLICT OF INTEREST STATEMENT

The authors declare no conflicts of interest

## Supporting information

Supporting Information S1

## Data Availability

The data that support the findings of this study are available on request from the corresponding author. The data are not publicly available due to privacy or ethical restrictions.
