# Supplementary material for: Breath of relief: Transforming pediatric asthma care with telemedicine‐guided exercises
Source: Clin Transl Allergy. 2025 Mar 24;15(3):e70049. doi: 10.1002/clt2.70049 (PMC11932885; doi:10.1002/clt2.70049)
Supplement: Supplementary file 1 — Supporting Information S1 [file CLT2-15-e70049-s001.docx]

**eMethodology**

1. **Assessment of asthma control**

**eTable S1.** Assessment of asthma control and asthma-related quality of life.

| Asthma Control Test (ACT)^12^ | ACT has been validated and comprises the following 5 items based on questions with responses scored from 1 to 5 points: (1) shortness of breath; (2) personal rating of asthma control; (3) use of asthma rescue medication; (4) limitations at work/school due to asthma; and (5) waking up because of asthma symptoms. The sum of the five-item points forms the ACT result, which ranges from 5 to 25 points, 25 representing the optimal asthma control. The sum of the scores allows asthma control to be categorized as follows: uncontrolled asthma (5–19 points), controlled asthma (20–24 points), and optimal disease control (25 points). |
| --- | --- |
| Asthma Control Questionnaire (ACQ)^13^ | The ACQ assesses 7 items, which include asking participants to recall their experiences in the last week and to respond to questions about night-time waking, symptoms on waking, activity limitations, shortness of breath, wheezing, required use of short-acting b2-agonists (SABA) for rescue, and FEV1 percent predicted before bronchodilator on a 7-point scale. All of these items are equally weighted, and the ACQ score is the mean of the 7 items and ranges from 0 (totally controlled) to 6 (severely uncontrolled). |
| GINA Symptom Control Tool (GINA-SCT)^4^ | GINA-Symptom Control Tool assesses 4 items, which include asking patients to recall their last 4 weeks' experiences and to respond to questions about daytime asthma symptoms more than twice/week, night-time waking, activity limitation, and SABA reliever for symptoms more than twice/week. This is a yes/no scale, and all of these items are equally weighted, and the score is the total of 4 items and ranges from 0 to 4 (0: well controlled, 1-2:parly controlled, 3-4:uncontrolled). |
| The Pediatric Asthma Quality of Life Questionnaire (PAQLQ)^14,15^ | The PAQLQ is a disease-specific questionnaire for children with asthma. It is intended to assess QoL in children between 7 and 17 years old. It includes 23 items in three domains, i.e., activity limitation, symptoms, and emotional functions. The activity limitation domain comprised five items relating to play, sports, and other daily activities. There were 10 items in the symptoms domain, including cough, wheezing, and nocturnal awakening. The emotional function domain contained eight related items such as being frightened, frustrated or feeling different, being irritable or worried, etc. Response to each item ranged from 1 (indicating maximum impairment) to 7 (no impairment at all). Patients were asked to recall the physical and emotional impairments that they experienced during the previous week. There are two types of questions. The first concerns the level of bothersome, and the second concerns the frequency of bothersome.  Each item was equally weighted. Scores of items belonging to each domain were summed and divided by the number of items of that domain. Scores on all items were summed and divided by 23, giving the overall score. Results were expressed as a mean score per item in each domain and for all 23 questions, ranging from 1 to 7. |

1. **Exclusion criteria** for severe asthma included (1) FEV1 < 60% of prediction on spirometry, (2) PEF variability > 30% at least twice during a one-week monitoring period, (3) use of short-acting bronchodilator medications at least four times daily, and/or (4) sustained daily oral steroid treatment. In addition, patients were excluded if they had any of the comorbidities preventing participation in the study (e.g., cardiac disease, musculoskeletal disease, chronic lung disease other than asthma, etc.), were asymptomatic in the past year, were treated with omalizumab or immunotherapy, did not have regular follow-up, had comorbidities that are significantly impacted by breathing exercises such as vocal cord dysfunction, inducible laryngeal obstruction, and/or dysfunctional breathing, and did not consent to participate in the study.
2. **Outcomes:**

**30snSTS (30-second sit-to-stand) test:** The functional capacity was assessed by the 30sSTS test. It has been reported that the 30sSTS test is a quick, valid, and alternative method for assessing functional capacity in both healthy young adolescents and children with chronic respiratory diseases (16, 40). The 30sSTS test was carried out according to the protocol described by Morita et. Al (17). A standard, armless chair with a height of 46 cm was used for testing. The chair was stabilized against a wall. Participants were asked to sit on the chair, come forward until their feet were flat on the floor, and fold their upper limbs across the chest. Then, they were instructed to stand all the way up until their legs were completely straight and sit back down until their bottom had clear contact with the chair as fast as possible for 30 s. Patients were not verbally encouraged during testing. A number of completed sit-to-stand repetitions in 30 s was recorded.

**BHT (breath-hold test):** The dyspnea perception was assessed by the BHT. Recent studies have shown that BHT is a simple, rapid, reliable and easily applicable method for assessing dyspnea perception in patients with asthma and other chronic respiratory diseases (18,19). The participants, in a sitting position, were instructed to breathe in (up to tidal volume) and out and at the end of the gentle exhalation to pinch their nose and hold their breath until the first involuntary movement of the respiratory muscles without encouragement (11). The breath-holding maneuver was repeated a maximum of 3 occasions separated by at least 2 min if there was >10% difference in time between the first and second test. The total breath-holding time was measured with a chronograph.

1. **Sample size.** The G*Power 3.1 (Universitaet Dusseldorf, Germany) program was used to determine the study's sample size (20). The sample size calculation was made on the result of a study with 95% power and a two‐tailed α level of 0.05 with a 0.77 effect size that investigated the effect of breathing exercises on asthma control tests in children with asthma (32). Based on the provided calculation, we estimated a sample size of a minimum of 45 participants for each group. Participants were included in the study by calculating at least a 10% increase in sample size, adjusting for the drop-out rate.

We contacted 125 patients who fulfilled the entry criteria; 13 did not consent to participate in the study, and the sample size was reduced to 56 subjects per group.

1. **Randomization and blinding.** The biostatistics researcher randomized 112 eligible patients, individually and independently according to age, gender, and BMI, from each other by drawing a lot indicating either the exercise group (EG) or the control group (CG). This was a single-blind trial, as the participants did not know their group, but the research assistants and physiotherapists could not be blinded because of the nature of the study.
2. **Exercise Program**

**eTable S2.** Exercise Intervention

| The Posture Exercise Program | The posture exercise program consisted of 2 phases: stretching exercises and relaxation positions. The stretching exercises consisted of three exercises which are shoulder elevation, shoulder circumduction, and scapular retraction exercise. All stretching exercises were performed as 3 sets of 10 repetitions at least 3 days a week and 2 times a day for 12 weeks. The relaxation positions consisted of 3 different relaxation positions which are standing, sitting and supine positions. Subjects were instructed to practice the most appropriate of these 3 positions for 10 to 15 minutes, both between exercises during the day and during periods of worsening symptoms for 12 weeks. The duration of one session of posture exercise program was 20 minutes which consisted of 10 minutes of stretching exercises and 10 minutes of relaxation positions. |
| --- | --- |
| The Breathing Exercise Program | The breathing exercise program consisted of three different breathing techniques which are pursed lip breathing, diaphragmatic breathing, and lateral costal breathing. The breathing exercises were combined with pursed lip breathing to ensure breathing control. All breathing exercises were performed as 2 sets of 5 repetitions 2 times a day at least 5 days a week for 12 weeks. Subjects were informed that they should take rest intervals of 5-6 tidal breaths between breathing exercises to prevent respiratory muscle fatigue and hyperventilation. The duration of one session of breathing exercise program was 20 minutes. |

1. **Statistical methods**. Primary outcomes (respiratory function test) were analyzed according to the intention-to-treat approach [21]. All randomized patients were included in the analysis except missing patients, who were 5 in EG and 8 in CG. Also, secondary outcomes were analyzed results based on a “per protocol” approach including only patients remaining in the study until 12 weeks. PASW Statistics 18 for Windows program was used for data input and statistical analysis. Mean, standard deviation and frequency were used to state results. Normality was checked. Independent sample t test was used for two independent group comparisons. Repeated measurements analysis of variance test was used for three dependent group comparisons, and paired sample t test was used for subgroup comparisons. Chi-square analysis was used for categorical data comparison. Statistical significance was accepted as p<0,05.

**eTable S3.** Baseline characteristics of study participants (Per protocol analysis).

|  | **Exercise Group**  **n=42 (%)** | **Control Group**  **n=41 (%)** | **Differences between groups *p* value** |
| --- | --- | --- | --- |
| **Age months, mean (SD)** | 158.35 (34.2) | 150.59 (29.74) | 0.274 |
| **Age group, n (%)**  8-12  12-18 | 15 (35)  27 (65) | 18 (43.9)  23 (56.1) | 0.374 |
| **Gender: female, n (%)** | 21 (50) | 16 (39) | 0.315 |
| **BMI**  <95 percentile  >95 percentile | 29 (69)  13 (31) | 28 (68.2)  13 (31.8) | 0.941 |
| **Sensitization, n (%)**  Non-sensitized  Sensitized (according to skin prick test)  House Dust Mite  Pollen  Other  Sensitization to multiple allergens  Uncertain due to dermographism | 12 (26.1)  30 (73.9)  8 (19)  2 (4.7)  1 (2.3)  18 (42.8)  1 (2.3) | 7 (17)  34 (83)  13 (31.7)  2 (4.8)  1 (2.4)  17 (41.4)  1 (2.4) | 0.641 |
| **Inhale steroid**  Regularly  If needed  **Inhale steroid and LABA**  Regularly  If needed | 14(33.3)  1(2.5)  23(54.7)  4(9.5) | 16(39.1)  1(2.4)  17(41.4)  7(17.1) | 0.340 |
| **Baseline Data**  30sSTS (mean, SD)  BHT (mean, SD)  ACT, (mean, SD)  ACQ, (mean, SD)  GINA-SCT, (mean, SD)  PAQLQ, (mean, SD)  **Total**  **Activity Limitation**  **Symptoms**  **Emotional Function**  Spirometry analysis (mean, SD)  **PEF %**  **FEV1 %**  **FVC %**  **FEV1/FVC %** | 18.84(3.93)  27.37 (15.25)  19.5 (3.92)  0.9 (0.74)  1.1 (1.26)  5.67 (1.1)  5.47 (1.23)  5.44 (1.22)  6.07 (1.2)  86.22 (15.27)  99.65 (15.4)  98.7 (22)  104.07 (9.67) | 17.7(3.58)  27.62 (10.65)  19.89 (3.43)  1.12 (0.7)  1.21 (0.99)  5.65 (0.84)  5.42 (1.15)  5.36 (1.01)  6.17 (0.94)  87.95 (14.16)  98.05 (11.5)  95.18 (12.66)  102.6 (9.38) | 0.272  0.152  0.604  0.194  0.666  0.910  0.864  0.771  0.714  0.599  0.597  0.380  0.489 |

**eTable S4.** Baseline characteristics of study participants (Intent to threat analysis).

|  | **Exercise Group**  **n=51 (%)** | **Control Group**  **n=48 (%)** | ***p* value** |
| --- | --- | --- | --- |
| **Age months, mean (SD)** | 153.15 (35.72) | 150.5 (29.38) | 0.785 |
| **Age group, n (%)**  8-12  12-18 | 23 (45)  28 (55) | 20 (41.6)  28 (58.4) | 0.840 |
| **Gender: female, n (%)** | 24 (47) | 18 (37.5) | 0.417 |
| **BMI**  <95 percentile  >95 percentile | 37 (72.5)  14 (27.5) | 33 (68.7)  15 (31.3) | 0.825 |
| **Sensitization, n (%)**  Non-sensitized  Sensitized (according to skin prick test)  House Dust Mite  Pollen  Other  Sensitization to multiple allergens  Uncertain due to dermographism | 13 (25.5)  38 (72.6)  12 (23.6)  3 (5.9)  1 (1.9)  21 (41.2)  1 (1.9) | 9 (18.7)  39 (79.3)  14 (29.1)  2 (4.1)  1 (2)  20 (41.6)  1 (2) | 0.421 |
| **Inhale steroid**  Regularly  If needed  **Inhale steroid and LABA**  Regularly  If needed | 18 (35.2)  1 (1.9)  28 (54.9)  4 (7.8) | 18 (37.6)  2 (4.1)  21 (43.8)  7 (14.5) | 0.685 |
| **Baseline Data**  30sSTS (mean, SD)  BHT (mean, SD)  Spirometry analysis (mean, SD)  **PEF %**  **FEV1 %**  **FVC %**  **FEV1/FVC %** | 18.84 (3.93)  27.37 (15.25)  85.74 (15.64)  98.92 (14.57)  99.44 (20.32)  102.78 (10.35) | 17.7(3.58)  27.62 (10.65)  89.77 (15.17)  100.71 (13.17)  97.67 (14.07)  102.71 (9.23) | 0.272  0.152  0.321  0.622  0.939  0.739 |

**eFigure S1. Change in spirometric analyses (n=99).**

A.Change in Forced Expiratory Volume in 1 second (FEV1) from baseline (BL) to 12-week follow-up (12-w FUP) p=0.742 in CG, p=0.01 in EG. B. Change in peak expiratory flow (PEF) measured by spirometry from baseline (BL) to 12-week follow-up (12-w FUP) p= 0.194 in CG, p<0.0001 in EG.

**eFigure S2. Comparison of Baseline and Post-Intervention FEV1 z-Scores Between Experimental and Control Groups Over 12 Weeks. p=0.161 in CG, p=0.014 in EG**
